# *Leishmania tarentolae* as an Antigen Delivery Platform: Dendritic Cell Maturation after Infection with a Clone Engineered to Express the SARS-CoV-2 Spike Protein

**DOI:** 10.3390/vaccines10050803

**Published:** 2022-05-19

**Authors:** Ilaria Varotto-Boccazzi, Micaela Garziano, Giulia Maria Cattaneo, Beatrice Bisaglia, Paolo Gabrieli, Mara Biasin, Alessandro Manenti, Diego Rubolini, Mario Clerici, Emanuele Montomoli, Gian Vincenzo Zuccotti, Daria Trabattoni, Sara Epis, Claudio Bandi

**Affiliations:** 1 Department of Biosciences, University of Milan, 20133 Milan, Italy; ilaria.varotto@unimi.it (I.V.-B.); giulia.cattaneo@unimi.it (G.M.C.); beatrice.bisaglia@unimi.it (B.B.); paolo.gabrieli@unimi.it (P.G.); claudio.bandi@unimi.it (C.B.); 2 Pediatric CRC ‘Romeo ed Enrica Invernizzi’, University of Milan, 20157 Milan, Italy; gianvincenzo.zuccotti@unimi.it; 3 Department of Biomedical and Clinical Sciences, University of Milan, 20157 Milan, Italy; micaela.garziano@unimi.it (M.G.); mara.biasin@unimi.it (M.B.); daria.trabattoni@unimi.it (D.T.); 4 Department of Pathophysiology and Transplantation, University of Milan, 20157 Milan, Italy; mario.clerici@unimi.it; 5 VisMederi, 53100 Siena, Italy; alessandro.manenti@vismederi.com (A.M.); emanuele.montomoli@vismederi.com (E.M.); 6 Department of Environmental Science and Policy, University of Milan, 20133 Milan, Italy; diego.rubolini@unimi.it; 7 Water Research Institute-National Research Council of Italy, IRSA-CNR, 20861 Brugherio, Italy; 8 IRCCS Fondazione don Carlo Gnocchi, 20148 Milan, Italy; 9 Department of Molecular and Developmental Medicine, University of Siena, 53100 Siena, Italy

**Keywords:** antigen vehicle, dendritic cells, SARS-CoV-2, antigen delivery, cytokines

## Abstract

Background: Protozoa of the genus *Leishmania* are characterized by their capacity to target macrophages and Dendritic Cells (DCs). These microorganisms could thus be exploited for the delivery of antigens to immune cells. *Leishmania tarentolae* is regarded as a non-pathogenic species; it was previously used as a biofactory for protein production and has been considered as a candidate vaccine or as an antigen delivery platform. However, results on the type of immune polarization determined by *L. tarentolae* are still inconclusive. Methods: DCs were derived from human monocytes and exposed to live *L. tarentolae*, using both the non-engineered P10 strain, and the same strain engineered for expression of the spike protein from SARS-CoV-2. We then determined: (i) parasite internalization in the DCs; and (ii) the capacity of the assayed strains to activate DCs and the type of immune polarization. Results: Protozoan parasites from both strains were effectively engulfed by DCs, which displayed a full pattern of maturation, in terms of MHC class II and costimulatory molecule expression. In addition, after parasite infection, a limited release of Th1 cytokines was observed. Conclusions: Our results indicate that *L. tarentolae* could be used as a vehicle for antigen delivery to DCs and to induce the maturation of these cells. The limited cytokine release suggests *L. tarentolae* as a neutral vaccine vehicle that could be administered in association with appropriate immune-modulating molecules.

## 1. Introduction

Upon invasion of vertebrate tissues, infectious microorganisms are phagocytosed by specialized cells, including Dendritic Cells (DCs). DCs are then responsible for the transportation of engulfed microbes to lymph nodes, where they act as antigen presenting cells, thus playing a fundamental role in the initiation of the adaptive immune response [1]. Therefore, whole microbial cells, such as those incorporated in attenuated or inactivated vaccines, are expected to elicit effective immune responses [2]. This result, however, has not always been achieved, possibly due to poor antigenicity with limited immune memory, or improper polarization of the immune response [3,4]. In addition, pathogenic microorganisms are not always suitable to be scaled-up for production of millions of doses, and safety issues should be considered for several microbial species [5]. As an alternative, non-pathogenic microorganisms, phylogenetically related with pathogenic ones, could be proposed as surrogates in vaccine development. For example, BCG (Bacillus Calmette-Guerin), a strain of *Mycobacterium bovis*, was developed as a vaccine against human tuberculosis [6], and it has also been engineered for the expression of antigens from other microorganisms. This approach suggests the possibility to use *M. bovis*, as well as other bacteria, as a candidate vaccine vehicle against a variety of diseases [7,8,9].

An eukaryotic microorganism that might be comparable to BCG, as a vaccine surrogate or as a vaccine vehicle, is *Leishmania tarentolae*. *L. tarentolae* is a parasite of reptiles and is not pathogenic to humans and other mammals. In addition to the phylogenetic distance from mammalian-associated *Leishmania* spp., and the lack of evidence of human cases of disease caused by this parasite, the non-pathogenicity of *L. tarentolae* also appears to be related with the absence of the A2 gene, which promotes visceralization in protozoa of the *Leishmania donovani* complex [10]. This protozoan has already been assayed in a murine model as a live vaccine against *L. donovani*, the aetiological agent of visceral leishmaniasis in humans [11]. In addition, similarly to BCG, *L. tarentolae* has also been manipulated for the expression of proteins from pathogenic *Leishmania* species, as well as from viruses, and tested as a living vaccine vehicle in murine models (e.g., [12,13,14,15,16,17]). A major feature that makes this microorganism interesting as a potential vaccine vehicle is that, upon its inoculation into mammalian tissues, it is expected to target DCs and other phagocytic cells [11,18]. This assumption is based on well-established knowledge concerning the biology of *Leishmania* species infecting humans and dogs, whose major niche for survival and replication is represented by phagocytic myeloid cells, including DCs, mainly in secondary lymphoid tissues [19]. Whether *L. tarentolae* actually targets DCs, after inoculation into subcutaneous tissues in mammals, is, however, far from being established.

In vitro studies are also needed to determine whether and how this microorganism activates DCs. Indeed, pathogenic *Leishmania* species tend to skew the immune response towards Th2, with alternative M2 macrophage activation, favoring parasite survival and multiplication within anergic phagocytes [9,19]. In susceptible hosts, this unbalanced immune response is associated with intense production of antibodies that are not protective against the infection and are instead responsible for severe pathological alterations associated with immune-complex disease [20].

Whether *L. tarentolae* behaves similarly to pathogenic species is still unclear. Initial results showed that *L. tarentolae* activates human DCs in vitro [11]; in addition, in murine models, this protozoan parasite induced Th1-type cytokine production, even in the absence of adjuvants [11]. However, most of the subsequent studies suggested the need to co-administer appropriate adjuvants, or to engineer *Leishmania* for the expression of immune-modulating molecules to activate a polarized Th1 response (e.g., [16,21]). Conversely, evidence for the expression of M2 markers in human macrophages, after stimulation with *L. tarentolae*, has also been reported [22].

Further investigations on whether and how *L. tarentolae* induces effective immune activation are thus urgently needed to guide the use of this organism as a vaccine against pathogenic *Leishmania* species, or as a vaccine vehicle against other infectious agents.

Herein, we present a study on monocyte-derived human DCs aimed at clarifying the response of these cells to live *L. tarentolae* infection, in terms of cytokine production and surface cellular markers, and to determine the actual uptake of these parasites by DCs. The study was conducted using both a wild-type strain of *L. tarentolae*, and the same strain engineered for expression of the spike protein from SARS-CoV-2, with the idea of using *L. tarentolae* as a vaccine vehicle against SARS-CoV-2.

## 2. Materials and Methods

### 2.1. Plasmid Construction and Leishmania tarentolae Transfection

SARS-CoV-2 spike protein was derived from the genomic sequence of the isolated virus “Severe acute respiratory syndrome coronavirus 2 Wuhan-86 Hi-1” released in January 2020 (number MN908947). The full-length sequence consists of 3720 nucleotides and presents a GSAS substitution at the furin cleavage site (residues 682–685) and a C-terminal 6xHis-tag. The codon-optimized DNA sequence of the spike gene was synthesized and cloned into the pLEXSY-sat2 vector (Jena Bioscience, Jena, Germany), which integrates into the chromosomal 18S rRNA (ssu) locus of the parasite. The plasmid was first cloned and propagated in *Escherichia coli*, then the parasite *Leishmania tarentolae* strain P10 was transfected with the linearized plasmid by electroporation using a Gene Pulser II (Bio-Rad Laboratories, Hercules, CA, USA), according to the manufacturer’s procedures. Engineered *Leishmania* strains (Lt-spike) were grown in a Brain Heart Infusion (BHI) liquid medium supplemented with porcine hemin (5 μg/mL; Jena Bioscience, Jena, Germany), penicillin 50,000 U/L, streptomycin 50 mg/L (Jena Bioscience, Jena, Germany) and nourseothricin (100 μg/mL; Jena Bioscience, Jena, Germany) at 26 °C in darkness under aerated conditions. For the weekly maintenance of the strains, cultures were diluted in fresh BHI medium twice. The expression of the target protein was confirmed by both Western blot analysis and immunofluorescence assays. In particular, cellular pellets of 10 clones were analyzed by Western blot using a SARS Coronavirus spike protein polyclonal antibody 1:3000 (Thermo Fisher, Waltham, MA, USA) and a horseradish peroxidase (HRP)-conjugated IgG anti-rabbit 1:30,000 (Thermo Fisher, Waltham, USA). The selected clones were also characterized for their membrane presentation of spike protein by immunofluorescence. Briefly, 2 × 10^5^ cells were applied to a glass coverslip and fixed in Phosphate Buffered Saline (PBS) containing 4% paraformaldehyde for 30 min. After 1 h of incubation with a blocking solution consisting of PBS supplemented with 1% Fetal Bovine Serum (FBS), the cells were stained for 2 h with SARS-CoV-2 spike antibody (2.5 µg/mL, GeneTex) and then for 1 h with Alexa Fluor 488-conjugated anti-rabbit IgG secondary antibody (5 µg/mL, Thermo Fisher). Finally, cells were stained with Concanavalin-A (20 µg/mL, Thermo Fisher, Waltham, USA) for 30 min. The stained cells were mounted with ProLong Gold Antifade Mountant with DAPI (Invitrogen, Waltham, MA, USA), covered with a coverslip and observed under a Confocal microscope high end for fast live imaging (Nikon A1-3D SIM). 

### 2.2. In Vitro Assays

#### 2.2.1. Dendritic Cell (DC) Differentiation

Whole blood samples were collected from nine healthy donors in Ethylene Diamine Tetra Acetic acid (EDTA) tubes (BD Vacutainer, San Diego CA, USA). Ethical clearance was obtained from the University of Milan Ethics Committee (number 14/22). Written informed consent was obtained after receiving information about use of their blood samples. The biological material was anonymized.

Samples were centrifugated at 1200 rpm for 10 min; plasma obtained was collected and stored at −20 °C for other analyses. Peripheral Blood Mononuclear Cells (PBMCs) were isolated by density gradient centrifugation on Ficoll (Cedarlane Laboratories Limited, Burlington, ON, Canada) and viable cells were counted with the automated cell counter ADAM-MC (Digital Bio, NanoEnTek Inc., Seoul, Korea). Monocyte percentage was determined by flow cytometry staining 2 × 10^5^ cells with CD45 conjugated with APC-A750 and CD14 PC7; cells were then analyzed by CytoFlex (Beckman Coulter, Pasadena, CA, USA). Subsequently, the monocyte population was enriched by negative selection of unlabeled target cells using a human Pan Monocyte Isolation Kit (Miltenyi Biotec, Bergisch Gladbach, Germany) according to the manufacturer’s procedure. The Pan Monocyte Isolation Kit is an indirect magnetic labeling system for the isolation of untouched monocytes from human PBMCs. Highly pure unlabeled monocytes are obtained by depletion of the magnetically labeled cells. After isolation, monocytes were stained as previously described to ascertain that the purity of the sample was above 80%. Monocytes were incubated at a concentration of 1 × 10^6^/mL in MO-DC differentiation medium (Miltenyi Biotec, Colonia, Germany). After three days of incubation at 37 °C, 5% CO_2_, an equal volume of medium was added to the cultures that were incubated for a further four days. DC differentiation was verified by flowcytometry based on morphological parameters (SS and FS) and the expression of CD1a, CD11c, CD45, DC-SIGN on Lineage- (LIN^−^: anti-CD3, -CD14, -CD16, -CD19 and -CD56) cells. DC purity was always above 90% (Supplementary Figure S1). 2 × 10^5^ cells were stained with CD1a conjugated with PCy5, CD11c conjugated with PC7, CD45 conjugated with APC-A750, DC-SIGN conjugated with PE and Lin conjugated with FITC (Società Chimici Italiani, Rome, Italy); cells were then analyzed with CytoFlex (Beckman Coulter, Pasadena, CA, USA). 

#### 2.2.2. In Vitro *L. tarentolae* DC Infection Assay

Human DCs were used to assess the internalization of recombinant *L. tarentolae* Lt-spike in comparison to *L. tarentolae* wild type strain (Lt-wt) and to characterize the immune polarization. On day 7, *L. tarentolae* cells were washed three times with sterile PBS, counted and resuspended in sterile RPMI-1640 medium. DCs obtained were harvested, counted and incubated without any stimulus (MED treatment) or in the presence of Lt-wt (Lt-wt treatment), or Lt-spike (Lt-spike treatment) at a multiplicity of infection (MOI) of 5 parasites per human cell for 4 h at 37 °C and 5% CO_2_ to allow the internalization of *L. tarentolae* [11]. Bacterial lipopolysaccharide (LPS, 1 mg/mL) (Sigma-Aldrich, St. Louis, MO, USA) was used as positive control (LPS treatment). After four hours, cells were washed and resuspended in RPMI 1640 medium (Euroclone, Milan, Italy) supplemented with 10% fetal bovine serum, 1% of L-glutamine (LG) and 2% pen-streptomycin (PS). Molecular and immunological analyses were performed after 24 h of incubation at 37 °C and 5% CO_2_.

#### 2.2.3. Phagocytosis and Immunofluorescence Assays 

To estimate the capability of *Leishmania* to be internalized by the DCs, after 4 h of incubation with the parasites, cells were collected, resuspended in 100 µL of PBS and cytocentrifuged (Cytospin, Hettich, Kirchlengern, Germany) for 5 min at 500 rpm on a slide and stained with Giemsa solution following the standard protocol (Sigma-Aldrich, St. Louis, MO, USA). The percentage of infected DCs and the number of parasites in each infected cell were determined with an optical microscope (100×); ten areas of two slides per treatment were used to determine these indices. Then, we tested the production of the spike protein by Lt-spike inside the DCs through an immunofluorescence assay. After the co-incubation with Lt-spike (see above), the DCs were fixed in 4% paraformaldehyde for 30 min, permeabilized with TritonX-100, stained for 2 h with SARS-CoV-2 spike antibody (2.5 µg/mL, GeneTex, Irvine, CA, USA) and then for 1 h with Alexa Fluor 488-conjugated anti-rabbit IgG secondary antibody (5 µg/mL, Thermo Fisher, Waltham, MA, USA). The stained cells were then treated with the dye nile red, 5 µg/mL for 20 min (9-diethylamino-5H-benzo[alpha]phenoxazine-5-one, ThermoFisher, USA), for the detection of intracellular lipid droplets; subsequently, the cells were mounted with ProLong Gold Antifade Mountant with DAPI (Invitrogen, Waltham, MA, USA), covered with a coverslip and observed under a Confocal microscope (Nikon A1-3D SIM; Nikon, Tokyo, Japan). 

### 2.3. Gene Expression Analysis in DCs

RNA from dendritic cells exposed to the four treatments was extracted by using the acid guanidium thiocyanate-phenol-chlorophorm method. The RNA was dissolved in RNase-free water and purified from genomic DNA with RNase-free DNase (RQ1 DNase; Promega, Madison, WI, USA). One microgram of RNA was reverse transcribed into first-strand cDNA in a 20 µL final volume containing 1 mM random hexanucleotide primers, 1 mM oligo dT and 200 U Moloney murine leukemia virus reverse transcriptase (Clontech, Palo Alto, CA, USA). cDNAs were analyzed using a PCR array that included a set of 21 optimized real-time PCR primers specified for 21 markers (listed in Table S1) plus two housekeeping genes on 96-well plates; the procedures suggested by the manufacturer were followed (Bio-Rad, Hercules, CA, USA). The “2^-delta Ct” method was applied to compare the values between groups.

### 2.4. Multiplex Cytokine Screening Panel

A 27-cytokine multiplex assay (cytokines listed in Table S1) was performed on DC culture supernatants in unstimulated condition and after 24 h of stimulation, using magnetic bead immunoassays (Bio-Rad, Hercules, CA, USA) and Luminex 100 technology (Luminex, Dallas, TX, USA) according to the manufacturer’s protocol. 

### 2.5. Statistical Analyses

To investigate differences among the four DC treatments (MED, Lt-wt, Lt-spike, LPS) in surface marker expression (assessed by flow cytometry), cytokine production (assessed with the Multiplex Cytokine Screening Panel) and cytokine expression (assessed by RT-qPCR), we relied on linear mixed models (LMMs) where we included donor identification code as a random intercept effect to account for repeated measurements performed on DCs derived from the same donor across different DC treatments. DC treatment was included in LMMs as a four-level fixed factor. Whenever residuals were significantly skewed, response variables were log_10_-transformed to improve normality (Table S1). Due to the high number of tested surface markers and cytokines, we applied a *p*-value correction (FDR correction using the Benjamini–Hochberg method [23]) within each of the three groups of predictors (surface marker expression, assessed by flow cytometry; cytokine production, assessed with the Multiplex Cytokine Screening Panel; cytokine expression, assessed by RT-qPCR). To investigate differences among DC treatments, *post-hoc* tests were performed only for markers and cytokines that showed a significant (*p* < 0.05) effect of DC treatment after FDR correction. LMMs were fitted using the SPSS 21.0 software.

## 3. Results

### 3.1. Lt-Spike Protein Analysis and Expression

**Figure 1:**
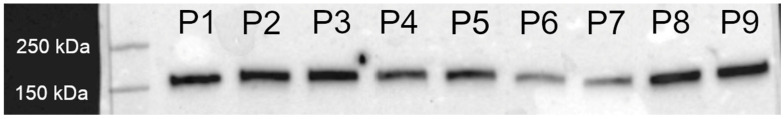
Evaluation of spike protein production in engineered *L. tarentolae*. Western blot analysis of Lt-spike expression in the pellet of nine clones of *L. tarentolae*. In all tested clones a band of approximately 180 kDa is appreciable using an anti-SARS-CoV-2 spike polyclonal antibody.

**Figure 2:**
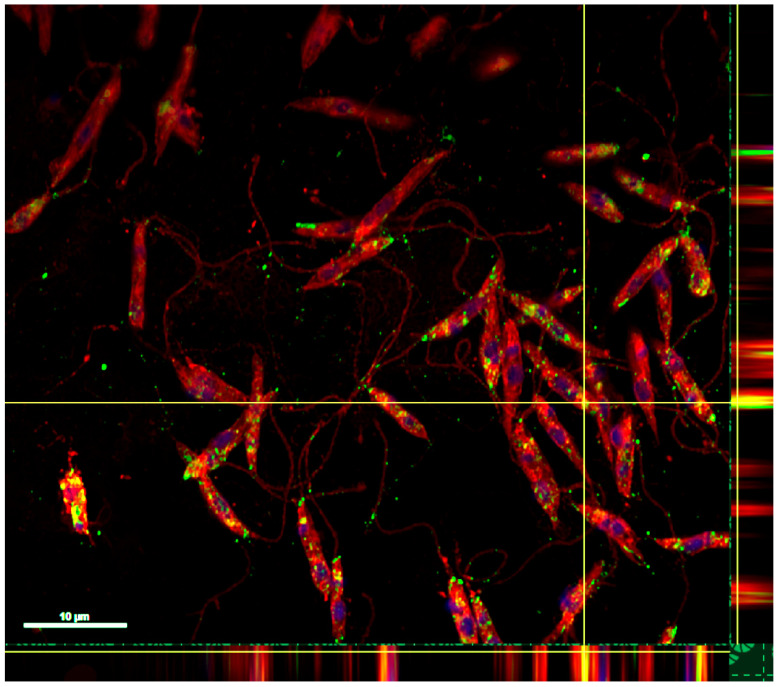
Spike protein production by engineered *L. tarentolae*. The presence of the spike protein on the parasite was evaluated by immunofluorescence staining using a SARS-CoV-2 spike antibody and the Alexa Fluor 488-conjugated anti-rabbit IgG secondary antibody. Green dots show the presence of the spike protein, while membranes are labeled in red using Concanavalin A staining. Yellow/orange dots are the result of the overlapping of the two signals (orthogonal projection) and are indicative of the colocalization of the spike protein on the membrane. The nucleus and kinetoplastid DNA was stained with DAPI (blue).

The expression vector encoding the entire sequence of the spike protein of SARS-CoV-2 virus (1240 aminoacids) was selected and used to transfect the parasite *L. tarentolae.* As shown by Western blot and immunofluorescence analyses, the spike protein was expressed both as a cytosolic and transmembrane protein. A band of approximately 180 kDa was visible in the pellet of all tested clones using a specific SARS Coronavirus spike protein polyclonal antibody (Figure 1; Supplementary Figure S2); the clone P9 was selected for the infection assays. An immunofluorescence assay was also performed using the combination of a specific anti-spike antibody and the Concanavalin-A, a cell surface dye, to investigate the presence of the protein at the surface of the *Leishmania*. In Figure 2, the green dots show the presence of the spike protein in the cytoplasm, while the colocalization of the protein and the external membrane signals are indicated by the yellow/orange dots.

### 3.2. Internalization of L. tarentolae by DCs

**Figure 3:**
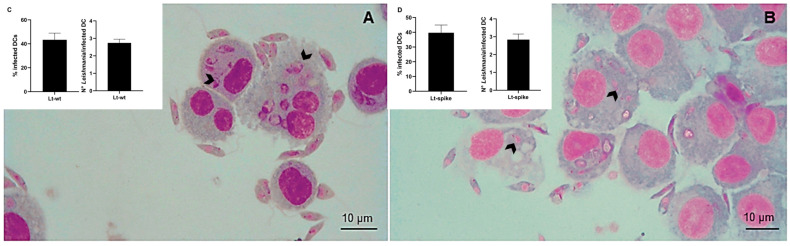
Internalization of *L. tarentolae* by dendritic cells after 4 h of incubation. Dendritic cells were incubated with Lt-wt (**A**) and Lt-spike (**B**) for 4 h at 1:5 ratio (DCs:*Leishmania*), then Giemsa smears were prepared and observed under a light microscope. Black arrows indicate the parasites inside the cells. The infection rate (percentage of infected DCs) and the mean number of parasites per infected cell (+SD) are reported for Lt-wt (**C**) and Lt-spike (**D**). Six hundred DCs were counted to determine these indices and the experiment was performed in duplicate.

**Figure 4:**
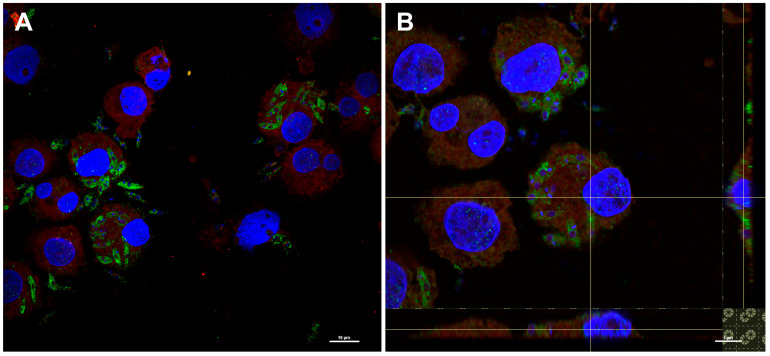
Spike protein production by engineered *L. tarentolae* after internalization in the dendritic cells. The presence of the spike protein on the parasite was evaluated by immunofluorescence after 4 h of co-incubation with human dendritic cells. After 4 h, cells were fixed and stained with SARS-CoV-2 spike antibody followed by Alexa Fluor 488-conjugated anti-rabbit IgG secondary antibody. (**A**) Green dots show the presence of spike protein in the cytoplasm of *Leishmania*, while the red signal (Nile red staining) shows the cytoplasm of the cells. The nucleus and kinetoplastid DNA was stained with DAPI (blue). (**B**) A magnification, with an orthogonal projection, showed the co-localization of the parasite and the cytoplasm of the cells as indicated by the presence of yellow/orange signals visible on the axes.

To demonstrate *L. tarentolae* parasite internalization by DCs, an experiment of co-incubation was performed. Monocyte-derived human DCs were exposed to parasites at a MOI of 5 (5 parasites:1 cell) for 4 h. This ratio and the time of incubation were defined on the basis of preliminary experiments (results not shown) and also considering published protocols [11]. At the end of the incubation, the cells were stained and the internalization of the parasite was determined. As shown in Figure 3, after 4 h of incubation, amastigote-like forms of *Leishmania* were visible inside the DCs; both the Lt-spike and Lt-wt strains were effectively phagocytosed by the cells, with no differences between the two strains. The percentage of infected DCs (infection rate) and the average number of parasites in each infected cell after 4 h of incubation were calculated and corresponded, respectively, to 43% and 2.74 for Lt-wt and 40% and 2.84 for Lt-spike. As reported in other studies (e.g., [18]), after 48 h, parasites no longer survived intracellularly: only a few amastigotes, partially degraded, were visible inside the cells with an infection rate of about 20% (Supplementary Figure S3). The presence of the spike protein on the parasite *L. tarentolae*, inside the DCs, was verified through an immunofluorescence assay (Figure 4; Supplementary Figure S4); Figure 4 shows that the spike protein was maintained at the surface (panel A) and in the cytosol of the protozoan (panel B), also after the internalization by the DCs. 

### 3.3. DC Maturation and Activation after L. tarentolae Infection

**Figure 5:**
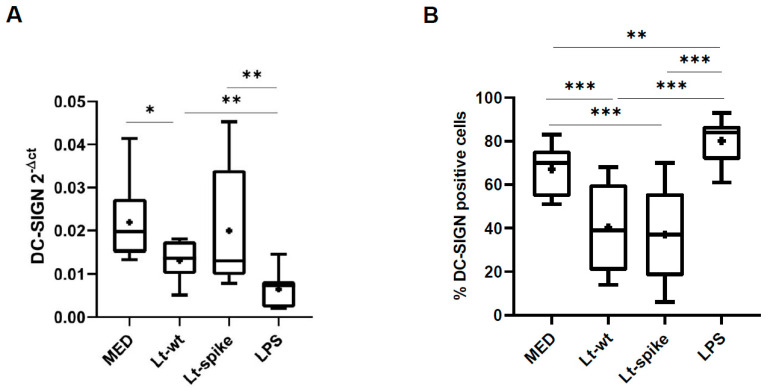
DC-SIGN expression in *L. tarentolae*-infected DCs. (**A**) DC-SIGN-specific mRNA expression was down-regulated upon internalization of Lt-wt. (**B**) The percentage of DC-SIGN expression following *L. tarentolae* infection was significantly reduced in DCs. DCs derived from healthy donors (n = 9). Box-plots show median, 25th and 75th percentiles, minimum and maximum values, while the symbol + indicates the mean value. Statistically significant differences at *post-hoc* tests are indicated; * *p* < 0.05; ** *p* < 0.01; *** *p* < 0.001. MED: untreated DCs.

A statistically significant reduction in mRNA DC-SIGN expression in DCs was observed following *Leishmania* infection (Lt-wt vs. MED treatment, Figure 5A; Table S1). This was confirmed by a significant reduction of DC-SIGN expression on DC surface in both Lt-wt and Lt-spike compared with MED treatment (Figure 5B; Table S1), suggesting a mechanism of uptake of the parasite by this receptor. Conversely, as expected (references [24,25]), the aspecific stimulus LPS resulted in a significant up-regulation of DC-SIGN expression compared with other cell treatments (Figure 5B).

**Figure 6:**
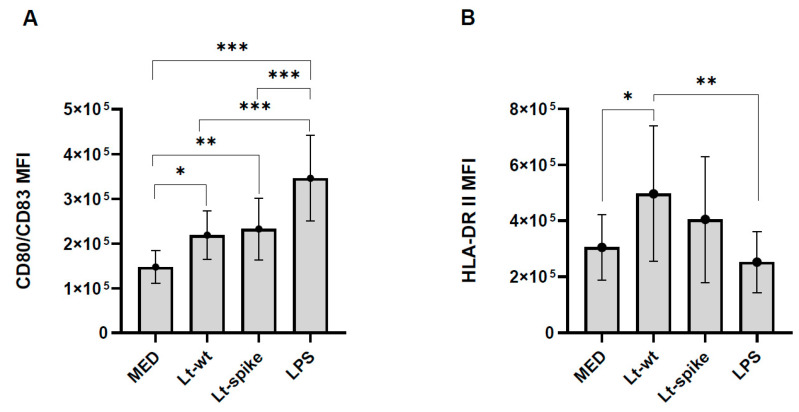
Maturation and activation of *L. tarentolae*-infected DCs. Mean Fluorescence Intensity (MFI) of CD80/CD83 (**A**) and HLA-DR II (**B**) was increased in *L. tarentolae*-infected DCs after 24 h of exposure. DCs derived from healthy donors (n = 9). Bars show mean ± SD values; statistically significant differences at *post-hoc* tests are indicated; * *p* < 0.05; ** *p* < 0.01; *** *p* < 0.001. MED: untreated DCs.

Following Lt-wt infection, DCs expressed significantly higher levels of CD80/CD83 and HLA-DR II compared to MED treatment (Figure 6). Lt-spike also increased the expression of CD80/CD83 compared to MED (Figure 6). No significant differences were observed between the two strains of *Leishmania* (Figure 6). 

**Figure 7:**
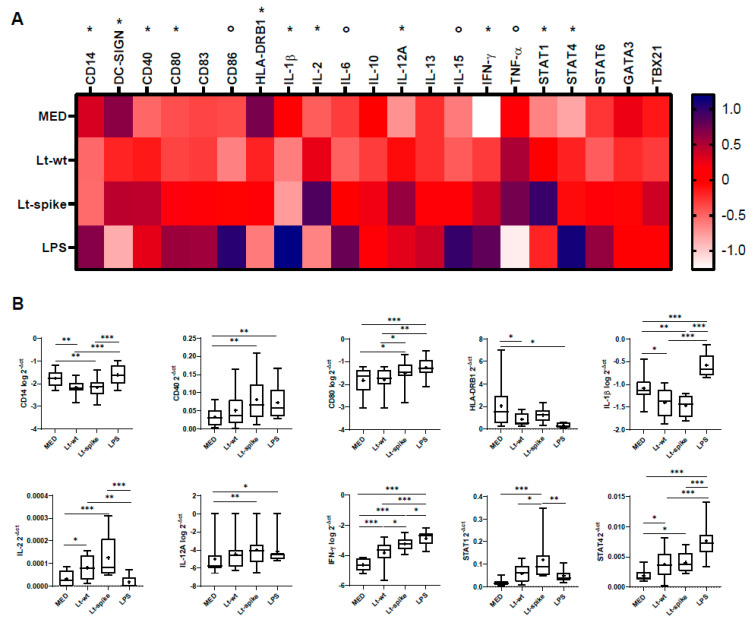
Immunological responses of *L. tarentolae*-infected DCs (RT-qPCR). mRNA expression of genes involved in the immune response in DCs exposed to Lt-wt and Lt-spike for 24 h. DCs derived from healthy donors (n = 9). (**A**) Gene expression (mean values) is shown as a color scale from white to blue (heatmap); variables, either on the original scale or log_10_-transformed (see Table S1), were standardized (mean = 0, SD = 1) to facilitate comparisons; * = at least one *Leishmania* cell treatment (Lt-wt/Lt-spike) significantly different (*p* < 0.05) from MED at *post-hoc* tests; ° = no *Leishmania* cell treatment significantly different (*p* > 0.05) from MED at *post-hoc* tests. (**B**) Box-plots (median, 25th and 75th percentiles, minimum and maximum values) of statistically significant markers, indicated in panel A with *. The symbol + indicates the mean value. Statistically significant differences at *post-hoc* tests are indicated; * *p* < 0.05; ** *p* < 0.01; *** *p* < 0.001. MED: untreated DCs.

Analysis of the DC expression profile following *L. tarentolae* infection revealed a mixed immune polarization outline (Table S1; Figure 7). On the one hand, compared to MED cells, *L. tarentolae* induced the up-regulation of CD40 (Lt-spike), CD80 (both Lt-wt and Lt-spike), IL-2 (both Lt-wt and Lt-spike), IL-12A (Lt-spike), IFN-γ (both Lt-wt and Lt-spike) and transcription factors STAT1 (Lt-spike) and STAT4 (both Lt-wt and Lt-spike) (Figure 7B). On the other hand, CD14 (both Lt-wt and Lt-spike), HLA-DRB1(Lt-wt) and IL-1β (both Lt-wt and Lt-spike) were down-regulated compared with MED cells (Figure 7B). Moreover, for CD80, IFN-γ and STAT1, Lt-spike showed a statistically significant up-regulation of expression profile compared with Lt-wt (Figure 7B). 

**Figure 8:**
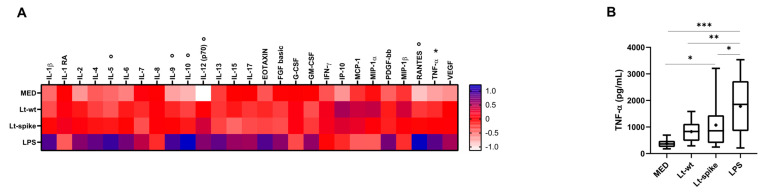
Immunological responses of *L. tarentolae*-infected DCs (multiplex cytokine screening). Secreted cytokine/chemokine concentration (pg/mL) obtained after 24 h of co-incubation of DCs with Lt-wt and Lt-spike. DCs derived from healthy donors (n = 9). (**A**) Cytokine/chemokine concentration (mean values) is shown as a color scale from white to blue (heatmap); variables, either on the original scale or log_10_-transformed (see Table S1), were standardized (mean = 0, S.D. = 1) to facilitate comparisons * = at least one *Leishmania* cell treatment (Lt-wt/Lt-spike) significantly different (*p* < 0.05) from MED at *post-hoc* tests; ° = no *Leishmania* cell treatment significantly different (*p* > 0.05) from MED cells at *post-hoc* tests. (**B**) Box plots (median, 25th and 75th percentiles, minimum and maximum values) of TNF-ɑ levels, indicated in panel A with *. The symbol + indicates the mean value. Statistically significant differences at *post-hoc* tests are indicated; * *p* < 0.05; ** *p* < 0.01; *** *p* < 0.001. MED: untreated DCs.

The analysis of *L. tarentolae* DC infected secretoma did not show any significant differences in cytokine production compared to uninfected DCs except for TNF-ɑ (Lt-spike vs. MED; Figure 8A,B; Table S1). However, although not statistically significant at *post-hoc* tests, a trend towards an increased production of IL-5, IL-9, IL-10, IL-12 and RANTES in *Leishmania*-infected cells compared to MED was observed (Figure 8A). As for the comparison between Lt-spike and Lt-wt, no significant differences were observed in the induction of cytokine production. As expected, LPS stimulation induced increased expression of almost all the soluble factor analyzed (Figure 8A).

## 4. Discussion

*L. tarentolae* has been investigated as a candidate vaccine, or as a vaccine vehicle, in in vivo studies on rodent models, following two major strategies. In a first approach, whole, living promastigotes were tested as vaccines against human pathogenic leishmaniae, either with [26,27] or without adjuvants [11]. In other studies, engineered strains of this microorganism were employed as a delivery platform, expressing antigens from a given pathogen (e.g., from pathogenic *Leishmania* spp. [21]) and/or immune modulating molecules [28]. In general, administration of *L. tarentolae* to rodent models in association with adjuvating molecules (e.g., CpG motifs, or pro-inflammatory proteins/peptides expressed by *L. tarentolae* itself [27,28]) determined a more effective immune response, compared to inoculation of un-adjuvanted promastigotes. Indeed, for several applications, particularly for the development of anti-*Leishmania* vaccines, the general consensus is that polarization of the immune response is required, on the M1/Th1 side to guarantee an effective activation of macrophages, with killing of intracellular parasites [9,29]. Knowledge on the type of immune-polarization determined by *L. tarentolae* is, however, limited.

The initial phase of the immune response is triggered by DCs that present antigens to T CD4+ lymphocytes and polarize these cells through the release of cytokines and the expression of co-stimulatory molecules. When investigating vaccine vehicles or surrogate pathogens as antigens, it is therefore crucial to determine the response of DCs, which then orchestrate the successive phases of the immune reaction. Our results demonstrate that *L. tarentolae* is actually engulfed by monocyte-derived DCs, similarly to human and canine parasitic leishmaniae (e.g., [30]). However, while DC maturation is normally impaired by pathogenic leishmaniae (reviewed in [19]), we observed that *L. tarentolae* infection induces a proper activation of these cells, consistent with an increase in the expression of molecules involved in antigen presentation (HLA-DR II and CD83 expression) and CD4+ T cell stimulation (CD80 expression). Moreover, exposure of DCs to both Lt-wt and Lt-spike determined a reduced expression of surface receptor DC-SIGN. DC-SIGN is a lectin receptor expressed on the membrane of phagocytic cells, including DCs and macrophages. It is involved in the binding of N-glycans which are displayed by viruses, bacteria and other pathogens and, in some cases, mediates their internalization [31,32]. The reduced presence of DC-SIGN on DC surface, after *L. tarentolae* infection, is coherent with the hypothesis that this parasite is phagocytosed by DCs through its binding with this lectin receptor. This observation agrees with those previously reported for other species of *Leishmania*, including *L. tarentolae* [11,33], and raises the possibility to exploit these parasites to interfere with the cycle of viruses, as suggested for HIV [34,35].

While in vivo studies have generally provided evidence for *L. tarentolae*’s capacity to skew the immune response toward Th1, mainly in association with adjuvating molecules, the immune-polarizing effect of this parasite on DCs has not so far been investigated. Our results show that the cytokines, induced by both Lt-wt and Lt-spike infection, are not entirely polarized towards a distinctive Th1 or Th2 profile. However, the expression of IL-2, IL-12, IFN-γ, together with that of specific transcriptional factors (STAT1 and STAT4) is suggestive of a Th1-like polarization, induced after parasite infection. Moreover, the induction of the Th1-like profile was more evident following Lt-spike infection, suggesting that the expression of this antigen, the spike protein, modifies DC activation. 

In summary, the two strains of *L. tarentolae* are able to target DCs, to be then phagocytosed, possibly following DC-SIGN binding, to induce DC maturation and a moderate immune polarization on the Th1 side. 

A major issue is whether engineered strains of *L. tarentolae*, modified for expression of heterologous proteins, maintain their capability to target and activate DCs, with a delivery of the antigen inside these cells. This issue is particularly relevant when dealing with protein antigens expressed at the surface of the *L. tarentolae*, which could potentially interfere with the molecular interactions of this microorganism with membrane receptors on DCs [36]. To this purpose, we have engineered a strain of *L. tarentolae* for the expression of the whole spike protein from SARS-CoV-2. After the selection of the transformed clone Lt-spike, which displayed the protein at the cellular surface, the assays performed on DCs confirmed that the modified strain maintained the propensity to be engulfed by DCs and to promote their maturation as revealed for the wild-type strain. 

## 5. Conclusions

*L. tarentolae* targets DCs, allows the delivery to these cells of the heterologous spike antigen of SARS-CoV-2 and induces their maturation. However, *L. tarentolae* can be considered a “nearly neutral” agent, able to induce a moderate expression and release of Th1 markers. Although this study was performed using an in vitro model, which lacks the complexity of in vivo systems (e.g., the interactions between different cell types and intercellular signaling phenomena) our results suggest that *L. tarentolae* is worthy of further investigations. It could indeed be developed as a neutral scaffold for the production and delivery of antigens in vaccination, to be associated with immune-modulating molecules, suitable to skew the immune response in the desired direction.

## Data Availability

The original contributions presented in the study are included in the article. Supplementary Material, further inquiries can be directed to the corresponding author.

## Supplementary Materials

The following supporting information can be downloaded at: https://www.mdpi.com/article/10.3390/vaccines10050803/s1. Figure S1. Gating strategy for DCs characterization. In the first gate doublets and dead cells were removed, then DCs were selected based on morphology. Finally, the gate of DCs was better characterized by expression of DC-sign, LIN-, Cd11c and CD1a. Figure S2. Western blot analysis of engineered *L. tarentolae* (Lt-spike strains) and *L. tarentolae* control strain (Lt-wt). Evaluation of the expression of spike protein in the pellet of five clones of Lt-spike (P5-P9) and Lt-wt (WT). In the recombinant clones a band of approximately 180 kDa is appreciable using an anti-SARS-CoV-2 spike polyclonal antibody, but not in the control strain (WT). Figure S3. Internalization and degeneration of *L. tarentolae* in human dendritic cells after 48 h of incubation. DCs were incubated with Lt-wt for 4 h at 1:5 ratio (DCs:*Leishmania*), then after two washes with PBS, cells were kept until 48 h at 37 °C. Giemsa smears were prepared and observed under a light microscope. Few amastigotes partially degraded are visible inside the cells indicated by blue arrows. DCs derived from healthy donors. Six hundred DCs were counted to determine these indices and the experiment was performed in duplicate. Figure S4. Immunofluorescence staining of the control sample *L. tarentolae* Lt-wt in dendritic cells. Lt-wt were internalized by DCs and stained using SARS-CoV-2 spike antibody. Absence of green fluorescence signals, characteristic of the spike protein production (see Figure 4), were observed following sample incubation with Alexa Fluor 488-conjugated anti-rabbit IgG secondary antibody. Red signal (Nile red staining) shows the cytoplasm of the cells. The nucleus and kinetoplastid DNA was stained with DAPI (blue). Table S1. List of tested surface markers and cytokines (RT-qPCR and Multiplex cytokine Screening Panel) and results of linear mixed models.
