# Supplementary material for: Leishmania tarentolae as an Antigen Delivery Platform: Dendritic Cell Maturation after Infection with a Clone Engineered to Express the SARS-CoV-2 Spike Protein
Source: Vaccines (Basel). 2022 May 19;10(5):803. doi: 10.3390/vaccines10050803 (PMC9144667; doi:10.3390/vaccines10050803)
Supplement: Supplementary file 1 [file vaccines-10-00803-s001.zip › vaccines-1655010-supplementary.pdf]

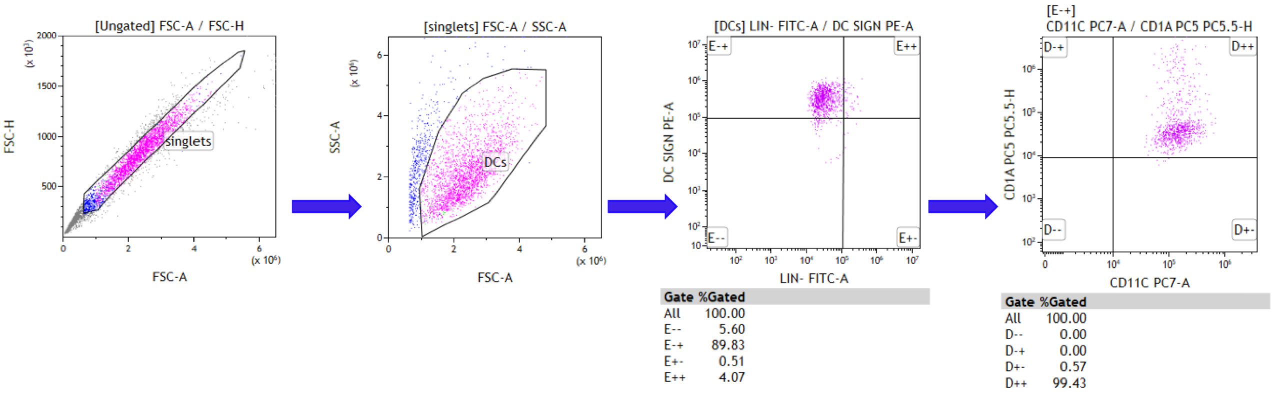

Figure S1. Gating strategy for DCs characterization. In the first gate doublets and dead cells were removed, then DCs were selected based on morphology. Finally, the gate of DCs was better characterized by expression of DC-sign, LIN-, Cd11c and CD1a.

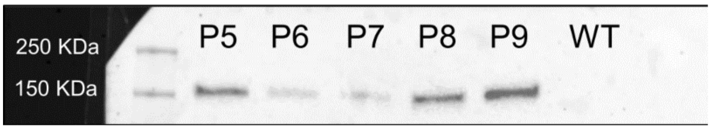

Figure S2. Western blot analysis of engineered *L. tarentolae* (Lt-spike strains) and *L. tarentolae* control strain (Lt-wt). Evaluation of the expression of spike protein in the pellet of five clones of Lt-spike (P5-P9) and Lt-wt (WT). In the recombinant clones a band of approximately 180 kDa is appreciable using an anti-SARS-CoV-2 spike polyclonal antibody, but not in the control strain (WT).

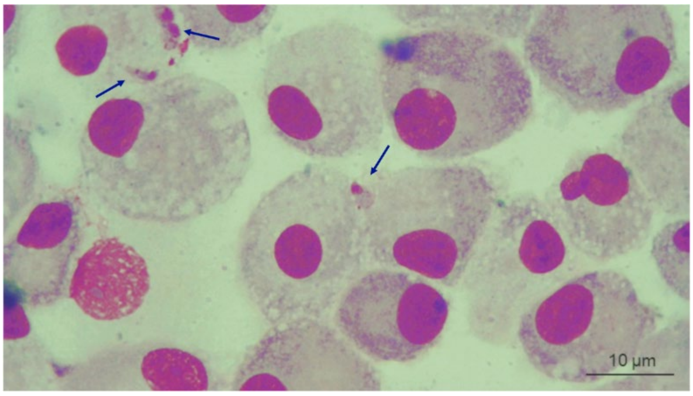

Figure S3. Internalization and degeneration of *L. tarentolae* in human dendritic cells after 48 h of incubation. DCs were incubated with Lt-wt for 4 h at 1:5 ratio (DCs:*Leishmania*), then after two washes with PBS, cells were kept until 48 h at 37 °C. Giemsa smears were prepared and observed under a light microscope. Few amastigotes partially degraded are visible inside the cells indicated

by blue arrows. DCs derived from healthy donors. Six hundred DCs were counted to determine these indices and the experiment was performed in duplicate.

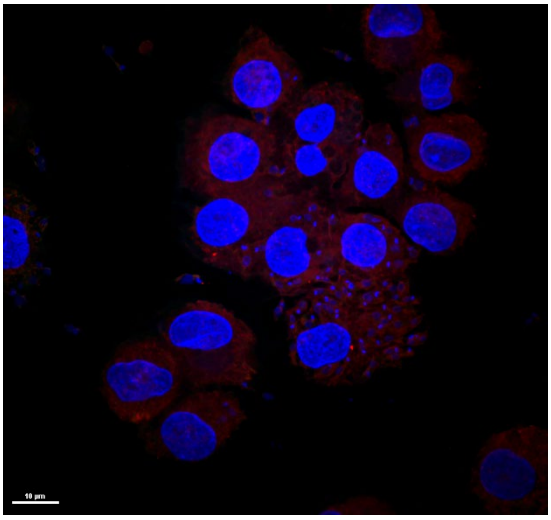

Figure S4. Immunofluorescence staining of the control sample *L. tarentolae* Lt-wt in dendritic cells. Lt-wt were internalized by DCs and stained using SARS-CoV-2 spike antibody. Absence of green fluorescence signals, characteristic of the spike protein production (see Figure 4), were observed following sample incubation with Alexa Fluor 488-conjugated anti-rabbit IgG secondary antibody. Red signal (Nile red staining) shows the cytoplasm of the cells. The nucleus and kinetoplastid DNA was stained with DAPI (blue).

Table S1. List of tested surface markers and cytokines (RT-qPCR and Multiplex cytokine Screening) and results of linear mixed models (including donor as a random effect) testing for differences between cell treatments (MED, Lt-wt, Lt-spike, LPS) in response variables. The sample size for each statistical test is reported in the rightmost column (minor differences in the number of measurements between analyses were related to failure to obtain a specific measurement for a given cell treatment). Degrees of freedom were estimated by the Satterthwaite's approximation. Tests that remained statistically significant ($p < 0.05$) after applying a correction for multiple testing (within each group of variables; FDR with Benjamini-Hochberg method) are highlighted in boldface. * = at least one *Leishmania* cell treatment (Lt-wt/Lt-spike) significantly different ($p < 0.05$) from MED cells at *post-hoc* tests; ° = no *Leishmania* cell treatment significantly different ($p > 0.05$) from MED cells at *post-hoc* tests; # = log_{10}-transformed variable.

Variable	F	d.f.	*p*	Sample size (measurements, donors)
Surface markers				
**CD80-CD83**	**17.980**	**3, 24**	**< 0.001**	**36, 9**
**CD80-CD83-HLA-DRII**	**3.554**	**3, 24**	**0.029**	**36, 9**
**HLA-DRII**	**3.588**	**3, 24**	**0.028**	**36, 9**
**DC-SIGN**	**37.299**	**3, 24**	**< 0.001**	**36, 9**
Cytokines (RT-qPCR)				
**CD14 #**	**14.293**	**3, 23**	**< 0.001**	**35, 9**
**DC-SIGN**	**5.882**	**3, 24**	**0.004**	**35, 9**
**CD40**	**4.072**	**3, 23.**	**0.018**	**35, 9**
**CD80 #**	**8.097**	**3, 23**	**0.001**	**35, 9**
CD83	2.955	3, 23	0.054	35, 9
**CD86**	**7.925**	**3, 24**	**0.001**	**35, 9**
**HLA-DRB1**	**4.079**	**3, 23**	**0.018**	**35, 9**
**IL-1β #**	**21.028**	**3, 23**	**< 0.001**	**35, 9**
**IL-2**	**10.671**	**3, 27**	**< 0.001**	**34, 9**
**IL-6**	**5.229**	**3, 23**	**0.007**	**35, 9**
IL-10 #	1.457	3, 23	0.252	35, 9
**IL-12A #**	**3.850**	**3, 23**	**0.022**	**35, 9**
IL-13 #	2.094	3, 22	0.130	34, 9
**IL-15**	**7,992**	**3, 23**	**0.001**	**35, 9**
**IFN-ɣ #**	**27.028**	**3, 19**	**< 0.001**	**32, 9**
**TNF-α #**	**19.789**	**3, 23**	**< 0.001**	**35, 9**
**STAT1**	**6.972**	**3, 23**	**0.002**	**35, 9**
**STAT4**	**15.333**	**3, 23**	**< 0.001**	**35, 9**
STAT6	2.457	3, 24	0.088	35, 9
GATA3	0.748	3, 25	0.534	35, 9
TBX21	0.735	3, 31	0.539	35, 9
Cytokines (Multiplex Cytokine Screening)				
IL-1β #	4.434	3, 24	0.013	36, 9
IL-1 RA	0.937	3, 24	0.438	36, 9
IL-2 #	1.229	3, 32	0.315	36, 9
IL-4	3.247	3, 32	0.035	36, 9

Variable	F	d.f.	*p*	Sample size (measurements, donors)
**IL-5°**	**6.383**	**3, 24**	**0.002**	**36, 9**
IL-6	3.182	3, 24	0.042	36, 9
IL-7 #	0.764	3, 32	0.523	36, 9
IL-8	0.180	3, 24	0.909	36, 9
**IL-9°**	**6.448**	**3, 24**	**0.002**	**36, 9**
**IL-10° #**	**21.016**	**3, 24**	**< 0.001**	**36, 9**
**IL-12 (p70)° #**	**6.513**	**3, 24**	**0.002**	**36, 9**
IL-13 #	1.453	3, 32	0.246	36, 9
IL-15 #	1.083	3, 32	0.370	36, 9
IL-17 #	1.082	3, 32	0.371	36, 9
EOTAXIN	3.911	3, 32	0.017	36, 9
FGF basic #	1.348	3, 32	0.276	36, 9
G-CSF #	0.637	3, 32	0.597	36, 9
GM-CSF #	0.867	3, 32	0.468	36, 9
IFN-ɣ	0.893	3, 24	0.459	36, 9
IP-10 #	1.711	3, 24	0.191	36, 9
MCP-1	2.372	3, 24	0.095	36, 9
MIP-1α #	1.565	3, 24	0.224	36, 9
PDGF-bb	4.106	3, 24	0.017	36, 9
MIP-1β #	1.431	3, 24	0.258	36, 9
**RANTES° #**	**18.931**	**3, 32**	**< 0.001**	**36, 9**
**TNF-α ***	**6.085**	**3, 24**	**0.003**	**36, 9**
VEGF	2.385	3, 32	0.088	36, 9
